# Signal Integration during T Lymphocyte Activation and Function: Lessons from the Wiskott–Aldrich Syndrome

**DOI:** 10.3389/fimmu.2015.00047

**Published:** 2015-02-09

**Authors:** Vinicius Cotta-de-Almeida, Loïc Dupré, Delphine Guipouy, Zilton Vasconcelos

**Affiliations:** ^1^Oswaldo Cruz Institute, Fiocruz, Rio de Janeiro, Brazil; ^2^UMR 1043, Centre de Physiopathologie de Toulouse Purpan, INSERM, Toulouse, France; ^3^Université Toulouse III Paul-Sabatier, Toulouse, France; ^4^UMR 5282, CNRS, Toulouse, France; ^5^Fernandes Figueira Institute, Fiocruz, Rio de Janeiro, Brazil

**Keywords:** Wiskott–Aldrich syndrome, T lymphocytes, actin cytoskeleton, motility, immunological synapse, signaling, transcription

## Abstract

Over the last decades, research dedicated to the molecular and cellular mechanisms underlying primary immunodeficiencies (PID) has helped to understand the etiology of many of these diseases and to develop novel therapeutic approaches. Beyond these aspects, PID are also studied because they offer invaluable natural genetic tools to dissect the human immune system. In this review, we highlight the research that has focused over the last 20 years on T lymphocytes from Wiskott–Aldrich syndrome (WAS) patients. WAS T lymphocytes are defective for the WAS protein (WASP), a regulator of actin cytoskeleton remodeling. Therefore, study of WAS T lymphocytes has helped to grasp that many steps of T lymphocyte activation and function depend on the crosstalk between membrane receptors and the actin cytoskeleton. These steps include motility, immunological synapse assembly, and signaling, as well as the implementation of helper, regulatory, or cytotoxic effector functions. The recent concept that WASP also works as a regulator of transcription within the nucleus is an illustration of the complexity of signal integration in T lymphocytes. Finally, this review will discuss how further study of WAS may contribute to solve novel challenges of T lymphocyte biology.

## General Presentation of the Wiskott–Aldrich Syndrome

The Wiskott–Aldrich syndrome (WAS) is an X-linked primary immunodeficiency (PID) characterized by the association of multiple clinical manifestations. Depending on disease severity, WAS patients may display susceptibility to viral, bacterial, and fungal infections, hemorrhages, eczema, multiple forms of autoimmune disorders, and susceptibility to develop hematological malignancies (1). The genetic defect at the origin of WAS has been identified 20 years ago (2), as mutations in the previously unknown gene encoding the WAS protein (WASP). This discovery has paved the way to the elucidation of WASP function. WASP expression is restricted to precursor and mature cells of the hematopoietic lineage (3), in which it promotes Arp2/3-dependent actin polymerization as an effector of the Rho GTPase Cdc42 (4, 5). Most of the defects associated with defective or abnormal WASP expression arise in mature hematopoietic cells rather than in their precursors. The nature of the mutations in the *WAS* gene and their consequence on the expression of WASP in hematopoietic cells correlate with the severity of the disease. The WASP-related pathologies include classical WAS (usually associated with defective WASP), X-linked thrombocytopenia (XLT, caused by residual point-mutated WASP), and X-linked neutropenia (XLN, caused by activating mutations). Over the last two decades, hundreds of studies have provided insights into how WASP regulates the activation and function of the different subsets of hematopoietic cells in which it is normally expressed (6). This has led to the notion that WAS results from the combination of a myriad of hematopoietic cell defects. In that respect, WASP deficiency illustrates how a single protein may participate in distinct functions in different cells (7). The ability of WASP to execute multiple tasks is linked to the fact that actin cytoskeleton remodeling is supporting many dynamic aspects of cell biology and that the different hematopoietic cells execute specific functions relying on a dynamic actin cytoskeleton.

The objective of this review is not to draw a comprehensive map of the functions executed by WASP in the numerous hematopoietic subsets. Instead, we will focus on the role played by WASP in T lymphocytes and will highlight how this line of research has contributed to our current understanding of signal integration during T lymphocyte activation and function. By attempting to link-specific WAS-related T lymphocytes defects to the clinical manifestations arising in WAS patients, we will also discuss how the study of WAS is helping us to understand the function of human T lymphocytes *in vivo*.

## Role of WASP in the Molecular Control of T Cell Activation

### Structural domains and molecular control of WASP activity

**Figure 1:**
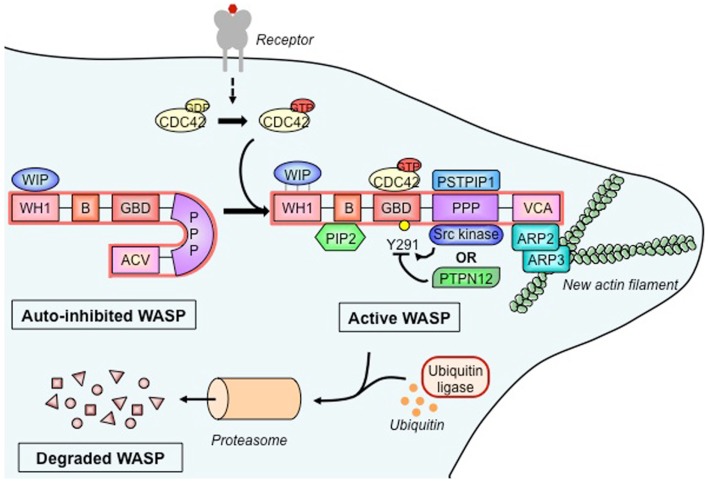
**Wiskott–Aldrich syndrome protein activation, molecular partners, and cytoskeleton remodeling**. In its basal state, the Wiskott–Aldrich syndrome protein (WASP) is auto-inhibited due to intramolecular interaction between its verprolin-homology domain–cofilin homology domain–acidic region (VCA) domain and its GTPase-binding domain (GBD). The association of WASP-interacting protein (WIP) with the WASP homology 1 (WH1) domain stabilizes the auto-inhibited form. Upon activation of a wide range of cell surface receptors (detailed in Figure 2), the Rho family GTPase cell division cycle 2 (CDC42) binds to the GBD, which causes the release of the VCA. Additional WASP activators include the proline–serine–threonine phosphatase-interacting protein 1 (PSTPIP1) and phosphatidylinositol-4,5-biphosphate (PIP2) that, respectively, bind the polyproline (PPP) domain and the basic (B) domain. Stability of active WASP is dependent on the phosphorylation status of tyrosine residue 291 (Y291), which is regulated by Src family tyrosine kinases and the tyrosine–protein phosphatase non-receptor type 12 (PTPN12). Active WASP binds, via its VCA, the actin-related protein 2 and 3 (ARP2-ARP3) complex and monomeric actin to produce a new actin branch. Dynamical actin remodeling relies on a balance between WASP activation and degradation, the latter being regulated by ubiquitination and mediated by the proteasome.

A close analysis of WASP reveals a molecular hub organized as a five-domain structured molecule (Figure 1). The first N-terminal domain, WASP homology 1 (WH1; also defined as EVH1, for ENA/VASP homology 1) binds to a proline repeat motif present in WASP-interacting protein (WIP) and mediates a molecular interaction regarded as critical for keeping a stable and auto-inhibited WASP conformation. The basic region also participates in the regulation of WASP conformational status, as it binds to the phosphoinositide PIP2 (phosphatidylinositol-4,5-biphosphate), which acts synergistically with the small GTPase Cdc42 to activate WASP (8). The GTPase binding domain (GBD), in a non-activated state of the WASP molecule, is found in an intramolecular hydrophobic link with the C-terminal VCA (composed by the verprolin-homology, central hydrophobic, and acidic regions) domain. Upon cell activation, GBD is the critical binding site for the active Cdc42-GTP, and such an interaction leads to a conformational change that releases the VCA domain, shifting auto-inhibited WASP to an active molecule. The proline-rich region contains several sites for binding of Src homology 3 (SH3) domain, playing a role as a central core for docking of various SH3 domain-containing proteins. The C-terminal VCA forms the actin-nucleating region of WASP. It binds both actin monomers and the Arp2/3 complex, which is composed of seven proteins that work together to assemble branches of actin that grow out of a pre-existing filament. Altogether, WASP binding regions provide a molecular hub for associations with several proteins that integrate distinct signals brought together during cellular WASP activity (7, 9).

In T cells, WASP is mainly found in a molecular association with WIP (Figure 1), a molecule regarded as the major controller of WASP activation (10). As shown by *in vitro* data, upon TCR stimulation, WIP recruits WASP to the immunological synapse and appears to facilitate Cdc42-mediated activation of WASP (11). On the other hand, *in vivo* studies showed that WIP favors WASP stability by working as a molecular chaperone (12). WASP activation is also under control of the phosphorylation at tyrosine residue 291 within GBD (Figure 1). Mediated by Src kinases, including Fyn and Lck, this phosphorylation acts synergistically with Cdc42 binding to activate WASP (13) and is likely to regulate various T cell functions (14). Therefore, dephosphorylation also controls WASP activity, possibly carried out by tyrosine-protein phosphatase non-receptor type 12 (PTPN12). As shown from mouse studies, this phosphatase interacts with WASP via proline, serine, threonine phosphatase-interacting protein 1 (PSTPIP1) and appears to mediate inhibition of WASP-induced immunological synapse formation (14). Additionally, the intramolecular GBD-VCA binding is regarded as a critical allosteric control of WASP activity, since mutations in GBD produce a constitutively active molecule that seems to directly affect myeloid and lymphoid cells, including lymphocyte number and proliferation, and increased T cell death and genomic instability, as found in X-linked neutropenia patients and in a mouse model (15–17).

Beyond the schematic representation of Figure 1, WASP-promoted actin nucleation appears to be regulated by interactive stoichiometry, as biochemical studies suggest that two WASP proteins provide a VCA dimer that delivers two actin monomers to the Arp2/3 complex (18). This process occurs upon WASP recruitment of Arp2/3 complex to inner cell membranes where they interact with actin filaments and, after an initial conformational activation of Arp2/3 complex, WASP proteins are dissociated and filament branching and growth can be carried out (19, 20).

Finally, WASP activity is also regulated through degradation (Figure 1). *In vitro* studies employing human T cells have shown that TCR ligation and tyrosine phosphorylation promote WASP cleavage by calpain and proteasomal degradation following Cbl E3 ligase-mediated ubiquitylation (12, 21, 22). Conversely, interaction with WIP leads to WASP protection (12), and disruption of the WIP–WASP partnership, due to mutations within the WH1 domain, could decrease the WASP cell pool, which forms the molecular basis for the several immune defects seen in WAS (23, 24). The relevance of WIP for WASP stability is supported by the recent demonstration that a patient genetically deficient in WIP showed clinical signs similar to those typical of WAS. In addition, WASP expression was corrected by the transduction of WIP into the T cells from this patient (24).

### Input signals leading to WASP activation

The multidomain nature of WASP allows a complex molecular partnership that integrates different pathways contributing to the T cell activation *scenario*. Indeed, WASP has been shown to be activated by the triggering of numerous cell leukocyte surface receptors including the TCR, costimulatory receptors, integrins, and chemokine receptors. The pathways linking TCR-driven signaling to WASP activation are closely related to its localization at the immunological synapse. *In vitro* studies employing mouse and human T cell–APC conjugates demonstrated that TCR stimulation induces a proximal phosphorylation cascade driven by Lck, ZAP-70, and Itk kinases (25, 26). Upon ZAP-70-induced phosphorylation of the transmembrane adaptor protein LAT, a complex of scaffold proteins such as Grb-2, SLP-76, and Gads are assembled at the inner face of plasma membrane. WASP can be recruited to this complex through binding of its proline-rich region to SH3 domain of Nck1 (or Nck2), a molecule that contains an SH2 domain that allows direct interaction to tyrosine-phosphorylated SLP-76. These interactions recruit WASP to lipid rafts and to the immunological synapse and also promote its activation by bringing it close to the guanine exchange factor Vav1 and to membrane-bound Cdc42 (11, 25–27). In parallel, *in vitro* biochemical assays revealed that phosphorylation of human WASP at tyrosine 291 by the Src kinase Fyn is regarded as an associated pathway for WASP activation (28).

Signals triggered through TCR engagement also induce conformational activation of integrins (inside-out signaling), as seen for the β2 integrin LFA-1 in the adhesive interaction with ICAM-1 on the APC surface. This high-affinity conformation of LFA-1 is organized in membrane microclusters that results in further adhesive strength for stable interaction and allows the intracellular β2-chain tail to interact with the actin cytoskeleton via actin-binding proteins, such as talin (29). Costimulatory receptors also appear to collaborate with TCR-triggered WASP activation and subsequent actin rearrangement at the immunological synapse. CD28 signaling activates Vav1 and NF-AT signaling as well as actin rearrangement in T cells, events known to improve TCR activation (30). CD2 stimulation leads to WASP proline-rich domain binding with PSTPIP1, a SH3-containing adaptor protein, which recruits WASP to the immunological synapse, a molecular event that might explain the impaired actin polymerization *in vitro* following CD2 stimulation of WASP-deficient mouse T cells (31). Chemokine receptors also bring important signal input to WASP activation. They are G protein-coupled receptors that promote *in vitro* chemotaxis of human and mouse T cells by employing a signaling cascade that includes activation of Vav and Cdc42 and, consequently, WASP regulation of T cell migration (32, 33).

A concept of multiple receptors involved in relaying signals, which activate WASP can also be supported, as a recent *in vitro* study revealed that about 120 transmembrane or membrane-associated proteins can trigger actin rearrangement through activation of WAVE (WASP-family verprolin-homologous protein) molecules (34). Adhesive and chemotactic stimuli seem to act cooperatively in controlling WASP activation, as suggested by the *in vitro* finding that fibronectin and CXCL12 promote additive Vav phosphorylation in human T cells, contributing for maximal migration (32). Costimulation signals also jointly work with other receptor-triggered actin-dependent process, as recently shown that *in vitro* NKG2D receptor costimulation regulates CXCL12-driven chemotaxis in human CD8^+^ T cells by increasing Cdc42 activity (35). Taken together, these findings point to a complex partnership constructing the signaling pathways from a coordinated response initiated by ligation of the TCR and its partner surface receptors toward localized WASP activation and subsequent actin remodeling.

### Decoding WASP activatory signals into cellular mechanisms

**Figure 2:**
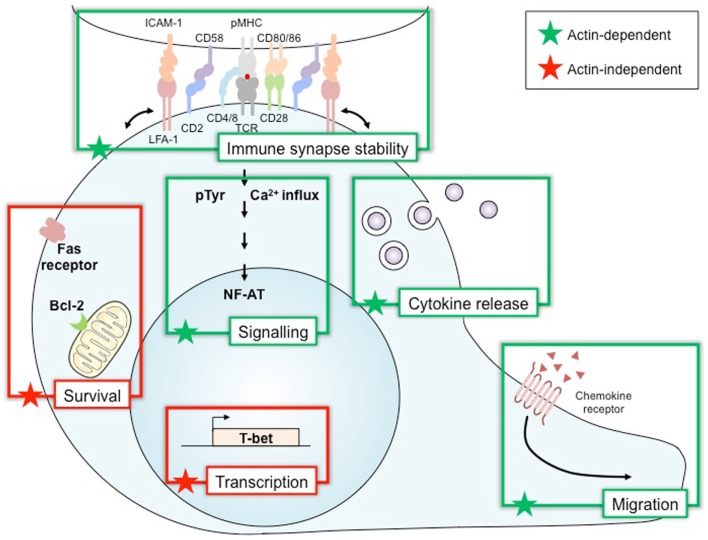
**Wiskott–Aldrich syndrome protein, a multiplex decoder of T cell activation and function**. Wiskott–Aldrich syndrome protein (WASP) is required for many functions in lymphoid immune cells. Many of these, such as immune synapse stability, signaling, cytokine release, and migration, relate to the role of WASP in regulating the polymerization of the actin cytoskeleton. Other functions of WASP depend on its activity in an actin-independent way as a transcription factor for T-bet and also in survival by controlling Bcl-2 and Fas expression. Bcl-2, B-cell lymphoma 2 protein; CD2 CD4/8 CD28 CD58 CD80/86, cluster of differentiation; ICAM-1, intercellular adhesion molecule 1; LFA-1, lymphocyte function-associated antigen 1; NF-AT, nuclear factor of activated T cells; pMHC, peptide/major histocompatibility complex; pTyr, phospho-tyrosine; T-bet, T-box transcription factor; TCR, T-cell receptor.

Wiskott–Aldrich syndrome protein activation plays a pivotal role in regulating actin dynamics following T cell stimulation. In fact, adhesion, polarization and motility, cellular events that underscore various main T cell functions arise as a consequence of regulated actin rearrangements in response to TCR engagement along with ligation of chemokine receptors and integrins (36). Nevertheless, WASP has been reported to drive T cell activities that can be distinctly divided accordingly to their dependence or independence on actin remodeling (Figure 2). In this context, cognate interaction of T cells with APC results in a stable contact that employs T cell-derived actin-rich membrane protrusions extending toward the APC. This T cell activation-induced structure is the cellular basis for immunological synapse formation (37). Molecular imaging studies with human T cells have elegantly showed that actin polymerization is dynamically responsive to TCR triggering, initiating at the TCR signaling site and further remodeled by WASP recruitment and proximal tyrosine phosphorylation events (38). Although we still miss a direct evidence for WASP regulation of actin flow-driven TCR microcluster dynamics (39), WASP activation in both human and mouse T cells has been demonstrated to be critical for the late and more stable interaction that forms the long-lived immunological synapse (40, 41). WASP deficiency seems to disorganize the immunological synapse *in vitro* as a consequence of impaired lipid raft clustering and scaffold signaling, resulting in abnormal localized actin polymerization (11, 27).

In parallel, as the cell secretion machinery is reoriented to this close contact site as well, this process also drives T cell effector functions associated with cytokine secretion (helper activity) and cytolysis (Figure 2). A mechanistic role can also be ascribed for WASP activity on these functions, since cytotoxic T cells from WAS patients show aberrant polarization of cytolytic granules toward the T cell–target cell contact area (42). In addition, WASP-deficient mouse T cells were shown to present impaired IL-2 production and cytokine secretion (43). Actin remodeling is also reported to regulate appropriate calcium and NF-AT signaling in T cells (44). Moreover, active WASP appears to play a critical role in both sustained calcium signaling (40) and NF-AT translocation (45), as IL-2 transcription and production are impaired in WASP-deficient mouse T cells (45, 46). Cell migration toward chemokine gradients guides important T cell effector activities (47). These chemotactic activities are actin-dependent mechanisms that employ active WASP, as chemokine-induced migration and homing of mouse T cells have been defined as reduced as a consequence of WASP deficiency (33, 48).

Wiskott–Aldrich syndrome protein activation is also reported to regulate Th1 cytokine gene transcription (Figure 2). T cells from WAS patients showed reduced IL-2 and IFN-γ transcripts associated to impaired NF-AT translocation and T-BET transcription following TCR/CD28 stimulation (49). Interestingly, the finding that WASP is present within human T cell nucleus and plays a pivotal role in regulating histone methylation at the *TBX21* promoter (50) reveals a regulated cytoplasmic-nuclear trafficking of WASP with direct effect on T-BET transcription and Th1 differentiation in an actin-independent manner (51).

Wiskott–Aldrich syndrome protein activation seems also to be involved in cell death receptor signaling (Figure 2), as some *in vitro* findings indicate that death-inducing signaling complex (DISC) assembly and Fas internalization induced by Fas signaling, including in human T lymphocytes, appears to be dependent on actin rearrangements (52). However, an actin-independent function for WASP in Fas signaling was suggested as well. Lymphocytes from WAS patients have increased CD95 expression and caspase-3 activity along with lower Bcl-2 expression (53, 54), which paralleled spontaneous accelerated apoptosis likely triggered via actin-independent mechanisms (54). Interestingly, TCR-stimulated CD4 T cells from WASP-deficient mice, which develop autoantibodies and glomerulonephritis, presented reduced apoptosis and FasL secretion (55). Altogether, these data indicate that WASP distinctly regulates spontaneous or TCR-induced cell death through mechanisms that include Fas/FasL signaling, controlling survival of normal T cells, and apoptosis of auto-reactive T cells. Nevertheless, impairment in both regulatory activities of WASP on T cell death can be related to immunodeficiency and autoimmunity in WAS patients.

These different WASP-driven cellular mechanisms might be regulated through distinct distribution of WASP and actin dynamics within subcellular compartments. Besides the localization in conjugated T cell–APC membranes and within the T cell nucleus, the targeting of WASP along with signaling molecules to the endocytic pathway points to an important subcellular localization of actin rearrangements following receptor engagement. TCR endocytosis triggered by antigen binding in human T cells was shown to be mediated by WASP activation upon binding to intersectin 2 (56). Moreover, *in vitro* studies demonstrated that human and mouse WASP bind to SNX9 (sorting nexin 9), a regulator of clathrin-mediated endocytosis (57). Such a binding seems to mediate both interaction with the PI3K pathway and CD28 endocytosis after TCR/CD28 costimulation. WASP seems to play an essential role in this process, as both CD28 endocytosis and actin rearrangements are impaired in WASP-deficient T cells (57). In spite of the critical signal transduction cascades evoked by input signals and the consequent WASP segregation into specific cell sites, how these subcellular compartments communicate to each other and which mechanisms control WASP trafficking (e.g., cytoplasm–nucleus) and its utilization from a common cellular pool are not yet established.

### Defective WASP expression and dysregulated T cell function

Cell adhesion, shaping, and migration are critical events for T cell development and function. As T cells develop, they emigrate from the thymus, enter in peripheral lymphoid organs, are activated after contacting cognate APC and, ultimately, work as helper, regulatory, or cytotoxic effectors. These cellular events are under the influence of actin dynamics. Migratory disturbances and aberrant T cell–APC interactions are regarded as the central dysfunctions, which underlies the immune defects reported in WAS patients (7). Several *in vitro* studies revealed that the defective interaction can be ascribed to the inability to stabilize immunological synapses, which is linked to defects in the assembly of focused signaling (11, 27, 40), distribution of high-affinity LFA-1 (58) and T cell–APC contact reformation (41).

Altogether, these data point out how signal inputs that link T cell activation to actin rearrangements are critical for proper T cell functions. Although mechanistic basis for regulation of the actin dynamics during T cell activation are not completely understood, WASP seems to be one of the critical molecules and to play a unique role in such a process, as regarded from the several abnormalities found in the WAS immunodeficiency (59). In fact, as discussed below, WASP deficiency renders major T cell subsets dysfunctional and T cell activation seems to be greatly impacted by impaired actin dynamics.

## WASP Deficiency Causes Selective Defects in T Cell Subsets

### T cell ontogeny and differentiation

Initial studies have revealed a status of a thymic hypoplasia at postmortem examination of patients with WAS (60). This suggests reduced thymic function and possible impairment of T cell development in this pathology. Depending on the cohorts of patients studied, T cell counts in early-aged patients were reported to be either normal or reduced, whereas a marked T cell lymphopenia in older patients was more systematically reported (61, 62). Recent studies have clarified the presence of TCR repertoire perturbations independently from the age of the WAS patients considered (63–65). These perturbations consist in reduced TCR diversity due to clonotypic expansions in memory CD4^+^ and CD8^+^ T cells.

Although selective pressure exerted by recurrent infections cannot be excluded, these data argue for a defect in thymic development and T cell generation in WAS patients. Additionally to central defects, there is evidence that reduced T cell survival could account for reduced lymphocyte numbers in the peripheral blood of WAS patients. Indeed, WAS T lymphocytes appear to be abnormally susceptible to apoptosis, potentially through up-regulation of the FAS-mediated cell death pathway (54). The T lymphocyte compartment of WAS patients was further investigated by studying the diversity of 24 TCRVβ subfamilies by CDR3 size distribution analysis (66). The results of this study clearly indicate that, in WAS patients above 15 years of age, the frequency of skewed TCRVβ subfamilies is abnormally high. This indicates an oligoclonal pattern of TCRVβ subfamilies usage in older patients with WAS, which might result from the progressive accumulation of antigen-specific T cell clones. Possibly, chronic antigenic stimulation consecutive to the inability to properly eradicate infectious agents could favor oligoclonality. Shrinkage of the T cell repertoire may also result from a failure to regenerate a diversified naïve T cell pool or from an abnormal susceptibility to apoptosis in the periphery. All in all, there is evidence for an anticipated aging of the T cell compartment in WAS patients that probably results from a combination of differentiation defects and overt stimulation in the periphery. As a result, the restricted T cell repertoire and biased T cell responses will ensure a less protective coverage against pathogens. In addition, this might favor the development of autoimmunity.

The critical role played by WASP in the homeostasis of human T lymphocytes is demonstrated by the *in vivo* selective advantage measured in patients with secondary *WAS* gene mutations allowing restoration of WASP expression (67). WASP re-expression and somatic mosaicism due to spontaneous *in vivo* reversion occurs in up to 11% of WAS patients (68) that in most of the cases is a phenomenon restricted to T lymphocytes. Reversion events restored T lymphocyte lineage characteristics as CD3-stimulated proliferation, actin rearrangement, WASP localization, cytokine production, and T cell receptor diversity (69–71). As a whole, these data suggests that selective advantage conferred by WASP expression has a major impact on T cell compartment. However, it is not clear whether the presence of somatic mosaicism correlates with clinical improvement (70, 72).

### Effector CD4^+^ T cells

**Figure 3:**
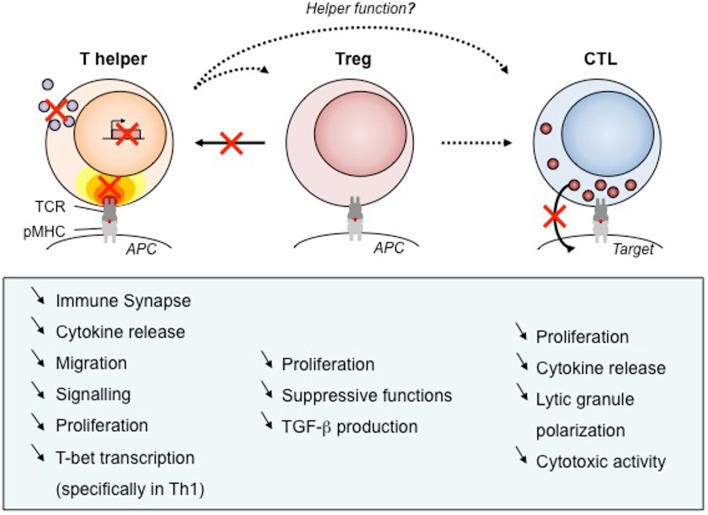
**Defects of T cell subsets and link to clinical manifestations**. As WASP is required for many functions, its absence results in defects of cellular function. We have listed those impairments for human T cell subsets that have been described in the literature. Black arrows show the function exerted by each T cell types, with dotted arrows to point out unknown WASP implication. Red crosses point out the most prominent impairments described in WAS T cells. APC, antigen-presenting cell; pMHC, peptide/major histocompatibility complex; TCR, T-cell receptor, TGF-β, transforming growth factor β.

Wiskott–Aldrich syndrome protein plays multiple roles in effector CD4^+^ T cells since it regulates motility, immunological synapse stability, signal integration, and cytokine production (Figure 3). T lymphocytes from WAS patients were originally found to display reduced chemotaxis in response to the T-cell chemoattractant stromal cell-derived factor (SDF)-1 (73). On the other hand, WASP-deficient CD4^+^ T cells display increased T cell motility upon encounter with non-cognate dendritic cells or B cells and reduced capacity to stop following antigen recognition (58). As an integrator of multiple signals arising from chemokine receptors, integrins, and the TCR, WASP may therefore modulate differently the motility of T cells depending on the context. As expected from the *in vitro* findings, WASP-deficient CD4^+^ T cells from the *Was*^−/−^ murine model display abnormal trafficking and scanning abilities. Indeed, the homing of WASP-deficient lymphocytes to Peyer’s patches is significantly impaired upon adoptive transfer into recipient mice, when compared to wild-type lymphocytes (48). Although not formally demonstrated, it is highly probable that CD4^+^ T cell motility defects contribute to the immunocompromised status of WAS patients.

Like for its role in T cell motility, WASP appears as a modulator of immunological synapse assembly. Rather than participating in the initial steps of immunological synapse assembly, WASP controls the stability of this complex structure (41). In particular, WASP is required for focused signal integration at the T cell/APC interface and sustained calcium signaling (40). It follows a sub-optimal T cell activation leading to decreased proliferative capacities (74). The selective defect in Th1 cytokine production observed in CD4^+^ T cell from WAS patients (75) was initially described as a consequence of abnormal calcium signaling and reduced nuclear recruitment of NF-AT-1 leading to defective T-BET induction (49). However, following shuttling into the nucleus of activated CD4^+^ T cells, WASP also controls T-BET activation directly as a transcription regulator (50). These two layers of control, at the synaptic and nuclear levels, over the transcriptional activation of Th1 cytokines in CD4^+^ T cells result in a marked Th1/Th2 imbalance (49). Although this has not been precisely evaluated, the WAS-related Th1/Th2 imbalance is expected to contribute to defective activation of antigen-specific T cell responses as well as to the pathogenesis of eczema. More in general, the lymphocyte defects in WAS patients at least partially account for the high susceptible to develop bacterial, viral, and fungal infections (76). A direct evidence for the contribution of T cell defective cytokine production to the immunodeficiency was provided by the administration to WAS patients of recombinant human IL-2 that appeared to be effective in reducing herpes virus infection (77).

Wiskott–Aldrich syndrome protein plays a key role not only during T cell development, activation, and functional maturation but also during the contraction phase following T cell reactivation. In particular, in absence of WASP, FasL release by CD4^+^ T cells is reduced and this is associated with decreased TCR-mediated apoptosis (55). Possible accumulation on CD4^+^ T cells after T cell reactivation might be associated with the frequent autoimmune complications occurring in WAS patients.

### Regulatory CD4^+^ T cells

Studies on T regulatory (Treg) cells from patients and from the *Was*^−/−^ murine model point to functional defects (Figure 3) that most probably contribute to the development of autoimmunity in WAS. WASP-deficient CD4^+^CD25^+^FOXP3^+^ regulatory T cells display defective proliferation, cytokine production, and suppressive functions (78–81). This was in particular demonstrated in a model of colitis induced by the transfer of CD45RB^+^ T cells in SCID mice, in which *Was*^−/−^ Treg cells were unable to prevent the development of the inflammatory process (80). Evaluation of natural Treg cell development clearly showed that WASP has a minor impact on the generation of Treg cells in the thymus (78–81). However, in the periphery, Treg cell suppressive function is impaired and can be partially rescued *in vitro* by treatment with IL-2 (78, 80). These data argue in favor of defective antigen-driven expansion and function. This is corroborated by the observation, in a case of spontaneous revertant mutation, that WASP-expressing Treg cells possess an *in vivo* selective advantage over WASP-defective Treg cells (79).

Autoimmune complications are a frequent manifestation in WAS patients, reaching an incidence of 72% in European and US populations (76, 82). The most common forms are autoimmune hemolytic anemia, cutaneous vasculitis, arthritis, and nephropathy. It is tempting to speculate that the multiple autoimmune manifestations in WAS patients arise as a consequence of a partial loss of self-tolerance due to defective Treg cell function. In parallel, intrinsic B cell dysfunction has been shown to contribute to the emergence of auto-reactive B cells (83, 84). Interestingly, activated Treg cells from *Was*^−/−^ mice display diminished ability to induce apoptosis in B cells when compared to wild-type ones. This impairment is associated with a significant reduction in granzyme B degranulation from those cells and, consequently, direct defective cytotoxicity against auto-reactive B cells (85).

Thus, the onset and development of autoimmunity in the context of WASP deficiency seems to reflect the inability of Treg cells to control the abnormal emergence of auto-reactive B cells. This reveals an important role of WASP in regulating key cellular activities for the control of auto-reactive cells and in the maintenance of self-tolerance.

### Cytotoxic CD8^+^ T cells

As in CD4^+^ T cell subsets, CD8^+^ T cells from WAS patients harbor defects in antigen-driven proliferation and cytokine production (49) (Figure 3). WAS CD8^+^ T cells have a profound impairment in the production of IL-2, IFN-γ, and TNF-α, which is due to defective gene transcription. The block in cytokine production is associated with reduced nuclear levels of NF-AT-1 and NF-AT-2. Defects in antigen-specific CD8^+^ T cell activation and cytokine production are also present in *Was*^−/−^ mice. In this model, the clearance of influenza A virus upon primary infection was normal, but the secondary response was impaired (86, 87).

Wiskott–Aldrich syndrome protein deficiency affects the cytotoxic activity of CD8^+^ T cells (42). In particular, WAS patient CTLs display a reduced cytotoxicity against tumoral B cell lines that can be rescued by restoring WASP expression by means of a lentiviral vector. This defect was associated to the lytic granule organization that appeared not to fully polarize toward the center of the CTL/tumor target cell contact area. Interestingly, cytotoxic function appears to be more profoundly affected in WASP-deficient NK cells than in WASP-deficient CTL (88, 89). A few reasons could account for this apparent difference, including a more pronounced requirement of WASP in NK versus CTL for the scanning and adhesion to target cells, the assembly of the lytic synapse, and/or the polarized delivery of lytic granules. Along those lines, studies on WASP-deficient NK cells have helped to characterize the assembly of a distinct NK cell lytic synapse (90, 91).

It has been proposed that combined defects in the cytotoxic function of CTL and NK cells could lower the level of immunosurveillance against tumors. Initial studies have reported tumor incidence in WAS patients to range from 13% (92) to 22% (76). This incidence might be underestimated given the increase of WAS patient life expectancy over the last decades. WAS-associated tumors are mainly leukemia, myelodysplasia, and B cell lymphoma (often EBV positive). WAS patients developing tumors belong to the highest risk group. The historical study reported that 2 years after diagnosis only 1 out of 21 patients had survived (92). The selective contribution of CTL defects to the abnormally high occurrence of tumors is difficult to establish in a context in which NK cells have profound defects in cytotoxic activity.

As mentioned, a significant proportion of patients with WAS present chronic viral infections, most commonly involving members of the herpes virus family (76). Surprisingly, little is known about the effects of WASP deficiency on antiviral immunity. Initial animal studies indicated increased susceptibility of *Was*^−/−^ mice to influenza infection, with implications on memory development when secondary viral challenge was evaluated (86, 87). More recently, *Was*^−/−^ mice were shown to be unable to clear an infection with the lymphocytic choriomeningitis virus (LCMV) and to declare increased immunopathology (93). This was associated with an intrinsic defect in the ability of CD8^+^ T cells to kill target cells presenting viral epitopes combined with a reduced priming due to the inability of DC to produce type I IFN. This is a demonstration that intrinsic defects of cytotoxicity in *Was*^−/−^ CD8^+^ T cells play a significant role in the control of viral infection *in vivo*.

### Additional T cell subsets

Wiskott–Aldrich syndrome protein appears to play a particularly important role in iNKT cells. Indeed, a profound reduction of circulating iNKT cells has been observed in WAS patients and seemed to correlate with severity of disease (94). In agreement, WASP is indispensable for the maturation of murine *Was*^−/−^ iNKT cells. In addition, WASP-deficient iNKT cells displayed defective homeostasis, homing, retention within peripheral lymphoid tissues and activation (94, 95). Further studies will be required to determine whether defective iNKT cell function affects pDC function.

Our knowledge about the role of WASP in T lymphocytes is still incomplete. In particular, many T lymphocyte subsets remain to be characterized in the context of WAS. Although γδ T cells have long been described to be abundant in the peripheral blood of WAS patients (96), their functional properties have not been tested. It will also be of interest to investigate the role of WASP in Th17 cells, since this T cell subset is associated to the development of inflammatory and autoimmune disorders (97). Interestingly, a recent study reported that exacerbated arthritis in *Was*^−/−^ mice was associated with an increase of Th17 cells and a decrease of regulatory T cells and B cells (98). This points to a possible role of WASP in Th17 homeostasis. Whether WASP modulates cytokine production in those cells also remains to be assessed. Unconventional Treg cell populations such as Tr1 cells and CD8^+^ Treg cells might also harbor specific defects in WAS and contribute to some clinical manifestations. Finally, WASP is also probably playing a role in the motility, activation, and function of T follicular helper cells, which play a crucial role in the maturation of antigen-driven B cell responses (99).

## Concluding Remarks

Although the deficiency in WASP is extremely rare in the human population, this protein has been one of the most studied pathology-associated molecules in T lymphocytes. Its role in integrating activation through multiple key receptors and in decoding activation into actin cytoskeleton remodeling has stimulated research interest. As a result, the elucidation of the roles played by WASP in T cells has contributed to our understanding of T cell activation and function.

Many key concepts that still remain to be elucidated are emerging from the study of WASP in T cells. WASP is probably activated concomitantly by several receptors. How is signal integration performed to tune local actin remodeling? WASP plays multiple actin-dependent and actin-independent roles within the cell. How is WASP compartmentalization regulated? Is there a coordination between different compartments such as the immunological synapse and the nucleus? WASP appears to play slightly different roles depending on the T cell subsets. Is this linked to the role of WASP in the fine-tuning of T cell activation or in controlling specific transcriptional programs?

New tools would help to answer these questions. In particular, a better visualization of WASP at work with high resolution would be crucial. A further definition of WASP partners in different subcellular compartments would also be required to elucidate the diversity of WASP function. In light of the role of WASP in tuning motility, in setting the threshold for antigen-driven stimulation and in regulating specific transcriptional programs, it is tempting to speculate that is has an impact on the developmental plasticity of CD4^+^ T lymphocytes.

To unravel the contribution of specific defects to pathology, finer models with one defect only at a time would be required. Then, a further challenge will be to consider that WAS does not just result from isolated T cell-intrinsic defects but from inter-related defects. In addition to the T cell intrinsic defects reviewed here, it is important to underline the extrinsic defects of T cell activation by APC (100, 101).

Beyond the scientific interest in elucidating the multifaceted role of WASP lies the prospect of improving the diagnosis and the treatment of WAS. As part of the development of gene therapy approaches, the understanding of the role played by WASP in setting the threshold for TCR-driven proliferation and IL-2 production has provided a robust read-out to compare the efficacy of WASP-encoding retroviral vectors (102, 103). This has also recently been used to assess restoration of T cell function in the WAS patients enrolled in the first gene therapy trials (63, 104). The follow-up of gene therapy treated patients as well as patients with secondary mutations leading to WASP re-expression in T cells (70) will also allow us to more precisely link WASP-dependent molecular control of T cell function to physiological immune responses.

## Conflict of Interest Statement

The authors declare that the research was conducted in the absence of any commercial or financial relationships that could be construed as a potential conflict of interest.
